# Effects of Thermal Acclimation on the Tolerance of *Bactrocera zonata* (Diptera: Tephritidae) to Hydric Stress

**DOI:** 10.3389/fphys.2021.686424

**Published:** 2021-09-03

**Authors:** Michael Ben-Yosef, Eleni Verykouki, Yam Altman, Esther Nemni-Lavi, Nikos T. Papadopoulos, David Nestel

**Affiliations:** ^1^Department of Entomology, Institute of Plant Protection, Agricultural Research Organization, Bet Dagan, Israel; ^2^Laboratory of Entomology and Agricultural Zoology, Department of Agriculture Crop Production and Rural Environment, University of Thessaly, Volos, Greece

**Keywords:** Tephritidae, *Bactrocera zonata*, desiccation resistance, temperature acclimation, nutritional reserves

## Abstract

Insects, similarly to other small terrestrial invertebrates, are particularly susceptible to climatic stress. Physiological adjustments to cope with the environment (i.e., acclimation) together with genetic makeup eventually determine the tolerance of a species to climatic extremes, and constrain its distribution. Temperature and desiccation resistance in insects are both conditioned by acclimation and may be interconnected, particularly for species inhabiting xeric environments. We determined the effect of temperature acclimation on desiccation resistance of the peach fruit fly (*Bactrocera zonata*, Tephritidae) – an invasive, polyphagous pest, currently spreading through both xeric and mesic environments in Africa and the Eurasian continent. Following acclimation at three constant temperatures (20, 25, and 30°C), the survival of adult flies deprived of food and water was monitored in extreme dry and humid conditions (<10 and >90% relative humidity, respectively). We found that flies acclimated at higher temperatures were significantly heavier, and contained more lipids and protein. Acclimation temperature significantly and similarly affected the survival of males and females at both high and low humidity conditions. In both cases, flies maintained at 30°C survived longer compared to 20 and 25°C – habituated counterparts. Regardless of the effect of acclimation temperature on survival, overall life expectancy was significantly shortened when flies were assayed under desiccating conditions. Additionally, our experiments indicate no significant difference in survival patterns between males and females, and that acclimation temperature had similar effects after both short (5–10 days) and long (11–20 days) acclimation periods. We conclude that acclimation at 30°C prolongs the survival of *B. zonata*, regardless of ambient humidity levels. Temperature probably affected survival through modulating feeding and metabolism, allowing for accumulation of larger energetic reserves, which in turn, promoted a greater ability to resist starvation, and possibly desiccation as well. Our study set the grounds for understanding the phenotypic plasticity of *B. zonata* from the hydric perspective, and for further evaluating the invasion potential of this pest.

## Introduction

Dispersion of insects to new environments and consequent geographic range expansions implies an evolutionary process in which the organism’s inherent phenotypic plasticity is selected by new environmental conditions (Beaman et al., 2016; Tay and Gordon, 2019). Adaptations to the environment usually build up gradually during natural dispersions of insect species to adjacent habitats or previously unoccupied environments (e.g., Ouisse et al., 2020). In other cases though, the process may be abrupt, such as during anthropogenic introductions of insects through long-range trading of commodities, or migration of animals carrying phoretic alien insect species (e.g., Meurisse et al., 2019). In both circumstances, the intrinsic physiological and behavioral plasticity of the species will determine its ability to respond to change in its natural habitat, or establish in novel environments (Bubliy et al., 2012; Little et al., 2020).

Acclimation (i.e., the habituation of the organisms’ physiology, metabolism, and/or behavior to the prevailing environmental conditions) is a form of plasticity that may confer resistance to environmental stress (Little et al., 2020; Terblanche and Hoffmann, 2020). Acclimation has been extensively studied in the past to examine response of insects to temperature stress (reviewed by Sgro et al., 2016; Terblanche and Hoffmann, 2020). Such studies show that thermal habituation can increase insect tolerance to acute heat or cold stress (Sgro et al., 2016). Similarly, short term acclimation to a desiccating environment can contribute to the resistance of insects to dehydration (Chown et al., 2011). Hence, understanding the effects of acclimation is a major step toward predicting the contribution of phenotypic plasticity to the potential distribution and invasion dynamics of plants and animals (Beaman et al., 2016; Bujan et al., 2021). This may be particularly relevant to organisms having negative economic or ecological impact, such as disease vectors, agricultural pests, or aggressive invaders, which disrupt the ecological stability of colonized environments (Lockwood et al., 2007).

Due to their reduced size and high surface to volume ratio insects are particularly prone to desiccation stress by transpiration of water through the cuticle and spiracle openings. Additionally, water may be lost through excretions of solid waste and fluids (reviewed by Weldon et al., 2019). To regulate their water balance, insects employ various physiological adaptations that enhance their resistance to desiccation or increase intrinsic water stores. These include secretion of hydrocarbons that reduce cuticular permeability to water or regulation of spiracle opening to decrease water loss through gas exchange (Gibbs et al., 1997; Kalra et al., 2017; Weldon et al., 2019). Such mechanisms may synergistically contribute to overall resistance to dehydration, and constitute a part of a plastic response driven by prior acclimation to a desiccating environment (Chown et al., 2011). Additionally, insects may generate water internally through catabolism of lipid and carbohydrate reserves, and thus better tolerate desiccation stress (i.e., metabolic water, as in e.g., drosophillid flies; Gibbs et al., 1997; Weldon et al., 2019 and referenced therein). Consequently, similarly to tolerance to other types of climatic stress (e.g., cold tolerance, Mohammadzadeh and Izadi, 2018; Raza et al., 2020), obtaining sufficient nutritional reserves may be imperative for the ability to resist desiccation (e.g., Gefen et al., 2006).

Nevertheless, depending on environmental conditions, higher respiration rates associated with metabolism may come at the expense of tolerance to extreme humidity (Kleynhans and Terblanche, 2011). For instance, under high temperature and relative humidity (RH) conditions, metabolism and respiration rates are not expected to drastically affect water balance in insects (Contreras and Bradley, 2011). Contrarily, when RH is low, gas exchange and metabolic regulation may come at a cost to the organism’s water balance, reducing its tolerance to extreme dry environments (Terblanche et al., 2008).

Temperature and desiccation stress often coincide for insects inhabiting xeric environments and their regulation may involve mutual adaptations. Indeed, the physiological and behavioral mechanisms activated to regulate metabolic rate and oxygen consumption in response to temperature and humidity in ectothermic organism are, in many cases, strongly related (e.g., regulation of gas exchange and heat and water balances in insects, Boardman and Terblanche, 2015; Thorat and Nath, 2018). However, little information is available regarding the effects of thermal acclimation on the ability of insects to withstand draught conditions and avoid dehydration (Kellermann et al., 2018; Thorat and Nath, 2018; Terblanche and Hoffmann, 2020), and the available data is often incomplete. For example, desiccation resistance was demonstrated to associate with temperature acclimation in Drosophilid (Kalra et al., 2017) and Tse-Tse flies (Terblanche and Chown, 2006), and to be mediated through excess secretion of cuticular lipids (Kalra et al., 2017). Similarly, when exposed to a desiccating environment, heat acclimated butterflies gained from reduced loss of body mass, possibly resulting from increased retention of water (Fischer and Kirste, 2018). However, in other cases (e.g., *Drosophila*: Gibbs et al., 1998, *Chirodica* beetles: Terblanche et al., 2005), thermal acclimation did not contribute to increased desiccation tolerance (see Chown et al., 2011 for other examples). Accordingly, thermal acclimation may affect the plasticity and tolerance of insects to extreme environmental humidity conditions, but such effects seem to be subject to variation (Chown et al., 2011; Terblanche and Hoffmann, 2020).

The peach fruit fly [*Bactrocera zonata* (Saunders); Diptera: Tephritidae], an invasive polyphagous pest of commercially-grown fruits, is progressively expanding its distribution in North Africa, the Middle East, and the Arabian peninsula in the past decades. Native to tropical southeastern Asia, the fly has invaded and established stable populations in Egypt during the early 1990s and has since dispersed to Libya, Sudan, and the desert of the Sinai Peninsula (Zingore et al., 2020). It has been intercepted on several occasions at the border between Israel and Egypt (EPPO Bulletin, 2005, 2010), and is currently frequently detected in the urban and suburban areas of Tel-Aviv, Israel (Gazit and Akiva, 2017). While no information currently exists on the source of the current outbreak in Tel Aviv area, it is suspected that the fly originates from the Israel-Egypt border and was unintentionally introduced to this urban area by humans.

The persistent detection of this fly in hot and dry desert regions, tropical habitats, and urban environments, where moderate temperatures and RH prevail throughout most of the year (Portnov and Safriel, 2004), point to a high phenotypic plasticity supporting its survival in both xeric and mesic environments. The current geographic distribution and the versatile climatic environments occupied by this species provide an excellent foundation to explore its plastic and adaptive responses to humidity stress.

The current paper is a first approach to characterize the ability of *B. zonata* to overcome hydric stress and adapt to xeric and humid environments. Here, we address the possible link between temperature acclimation and tolerance to humidity, and examine if temperature could trigger enhanced resistance to humidity stress. Accordingly, we exposed flies to different thermal regimes and subsequently assayed them for survival at humid and dry environments. As metabolism is modulated by temperature, we additionally examined the effect of acclimation temperature on weight and nutritional reserves of the fly, and tested for possible correlations with survival.

## Materials and Methods

Fly origin and maintenance: *B. zonata* pupae were obtained from a laboratory colony established in 2012 from flies collected in the area of Tel Aviv, Israel, and routinely maintained at the quarantine facility of the Plant Protection and Inspection Services Laboratories in Bet-Dagan, Israel (see Gazit and Akiva, 2017 for details). The colony’s breeding population was last refreshed with wild flies in 2019. Following emergence, 1–2-day-old adults were separated by sex into 30 cm × 30 cm × 30 cm cubic screen/Plexiglas cages supplied with a standard diet of sugar and hydrolyzed yeast (3:1 ratio by weight; Tsitsipis, 1989 and references therein) and water. Male and female cages were maintained at three constant acclimation temperatures (20, 25, and 30°C), ambient humidity (50 ± 10% RH), and photoperiod of 16:8, hours of light:dark, respectively. Adult survival in extreme dry and humid environments (see following section) was subsequently determined for the three thermal acclimation regimes following 5–10 and 11–20 days of acclimation (short and long acclimation period, respectively). The use of constant temperature regimes to study acclimation has been previously suggested as unrealistic ecologically (Terblanche et al., 2010; Terblanche and Hoffmann, 2020). Nevertheless, herein, we decided to use such a protocol in order to maximize the expression of habituation traits in our experiment.

### Bioassays

Following acclimation, randomly sampled flies were weighed to the nearest 0.1 mg, individually confined to aerated 5 ml glass vials, and introduced into two transparent humidity chambers. The humidity in each chamber was preconditioned to 5 ± 3% RH (extreme dry) or 95 ± 3% RH (extreme humid) using dehydrated silica gel or a beaker with water placed inside the chamber, respectively. Assays continued uninterruptedly for 4 days at 25°C and 16:8 light:dark cycle, during which mortality was monitored visually every 2 h (during day time) or 10–12 h (during night time), without compromising the atmosphere within the chambers. Humidity levels in each chamber were constantly recorded using HOBO U12 data loggers (Onset, MA, United States), and verified to remain within the limit of the preset values. When possible three replicates were conducted for each combination of acclimation temperature, acclimation duration, and humidity regimes (*n* = 19 groups). However, due to shortage in flies, some combinations included two replicates (*n* = 5 groups; overall *n* = 24 groups). For each group, a replicate consisted of 8–10 individuals of each sex (overall *n* = 18–30 flies in each group, total *n* = 646 flies).

### Chemical Analyses

We evaluated the effect of acclimation temperature on the accumulation of lipid and protein reserves in males and females by using the colorimetric phospho-vanillin and Bradford assays, (respectively) as previously described (Nestel et al., 2004). Assays were performed on individual, 18 day old flies (*n* = 7–15 flies per treatment) sampled from each acclimation group. Briefly, for lipid quantifications, flies were homogenized in 100 μl of Na_2_SO_4_ (2%) after which lipids were extracted by adding 1,400 μl of Chloroform:Methanol (1:1 by volume, respectively) to each sample. Solvents were later evaporated from a 250 μl aliquot of the supernatant, and the remaining residues were reacted with 250 μl of H_2_SO_4_ at 90°C for 10 min. Subsequently, 30 μl duplicates of the resulting sample were mixed with 270 μl of Vanillin reagent (600 mg vanillin dissolved in 100 ml distilled water and 400 ml 85% H_3_PO_4_), and the optical density was recorded at 530 nm. Total lipid amounts were calculated using a standard curve generated with Triolein.

Protein analyses were performed by individually homogenizing each fly in 500 μl of phosphate buffered saline. Subsequently, 10 μl duplicates of the resulting supernatant were reacted with 200 μl of diluted Bradford reagent (Biorad, United States), and absorbance was recorded at 595 nm. Total Soluble protein amounts were extrapolated from standard curves generated with bovine serum albumin.

### Statistical Data Analysis

Cox proportional hazards models were used to test the effects of acclimation temperature, sex, and RH on survival of the flies in each of the two acclimation periods. Additionally, the risk of death was computed for each group. These models were adjusted for the body weight of the flies at the beginning of the assay, and accordingly, the predicted median survival times were computed by using marginal estimates at the mean weight. During model construction, two-way interactions were examined and confirmed as non-significant. Accordingly, these were not included in the final analyses.

Generalized linear models were used to examine the effects of acclimation temperature and sex on the fresh weight of flies for each of the two acclimation periods. A similar model was employed to examine the effects of acclimation temperature and sex on lipid and protein contents of 18-day old flies. Within these models, means of different groups were separated by Tukey’s HSD comparisons.

Means and SEs are reported. Values of *p* less than 0.05 were considered statistically significant. Statistical analysis was conducted using the JMP statistical package (SAS, Cary, NC, United States).

## Results

### Effect of Acclimation Temperature on Weight Gain

**Figure 1:**
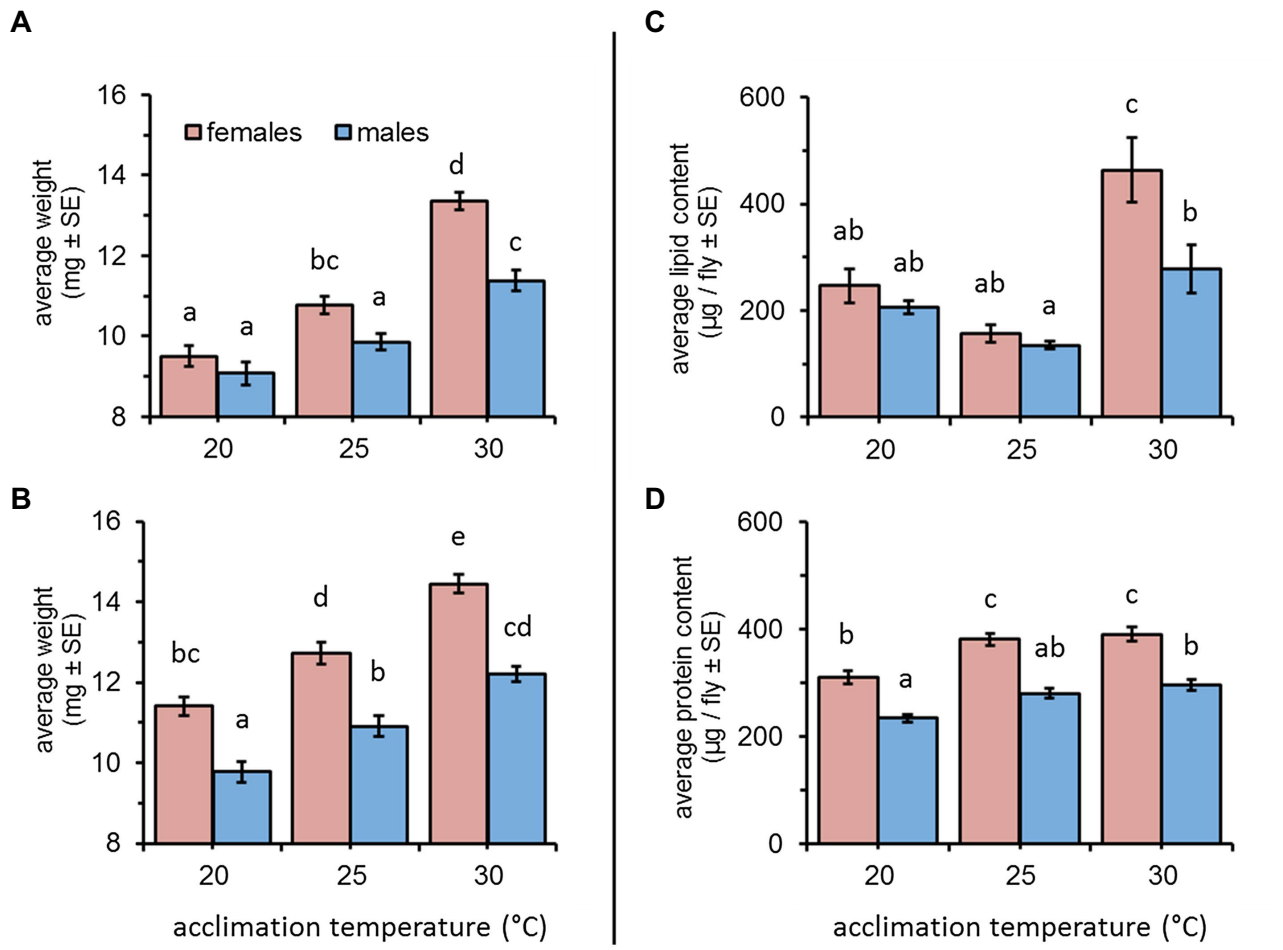
Average weight, lipid, and protein content of female and male *Bactrocera zonata* acclimated at 20, 25, and 30°C constant temperature regimes. Weight was recorded following a short (5–10 days, **A**) and long (11–20 days, **B**) acclimation period (*n* = 37–59 flies in each group). Lipids **(C)** and proteins **(D)** were quantified in individuals acclimated for 18 days (*n* = 7–15 flies in each group). Means separated by different letters are significantly different (*post hoc* Tukey HSD comparisons).

Weight gain was significantly affected by acclimation temperature [*F*(2, 347) = 83.87, *p* < 0.001] and sex [*F*(1, 347) = 31.45, *p* < 0.001] when flies were acclimated for a short (5–10 days) period. Overall, elevated temperatures were associated with a significant increase in weight for both males and females (Tukey HSD comparisons, *p* < 0.05, Figure 1A). Depending on temperature, females weighed 9.5 ± 0.25, 10.77 ± 0.21, and 13.36 ± 0.21 mg (at 20, 25, and 30°C, respectively). Males were similarly affected by temperature and weighed 9.07 ± 0.28, 9.85 ± 0.20, and 11.38 ± 0.26 mg when acclimated at 20, 25, and 30°C, respectively). Sex had a significant effect on weight only for 25 and 30°C – acclimated flies, resulting in a significant interaction between sex and acclimation temperature [*F*(1, 347) = 5.36, *p* = 0.0051, Figure 1A].

Similar effects were recorded for flies acclimated for a period of 11–20 days (acclimation temperature: *F*(2, 297) = 75.75, *p* < 0.001); sex: [*F*(1, 297) = 94.07, *p* < 0.001]. Here, also weight was significantly increased at elevated temperatures (Tukey HSD comparisons, *p* < 0.05, Figure 1B). Females weighed 11.40 ± 0.22, 12.71 ± 0.27, and 14.44 ± 0.23 mg, and males weighed 9.77 ± 0.25, 10.91 ± 0.25, and 12.19 ± 0.18 mg when acclimated at 20, 25, and 30°C (respectively). Females were consistently heavier than males, regardless of acclimation temperature (sex × acclimation temperature: *F*(2, 297) = 1.02, *p* = 0.360, Figure 1B).

### Effect of Acclimation Temperature on Lipid and Protein Reserves

Lipid contents of flies acclimated for 18 days were significantly affected by acclimation temperature [*F*(2, 68) = 22.83, *p* < 0.001] and sex [*F*(1, 68) = 8.86, *p* = 0.004]. However, the effect of sex differed between the three acclimation temperatures [acclimation temperature × sex: *F*(2, 68) = 3.45, *p* = 0.037]. When acclimated at 20°C and 25°C, the lipid contents of males and females remained relatively low (males: 205.34 ± 12.31 and 134.42 ± 6.93; females: 246.20 ± 31.97 and 156.55 ± 17.11 μg lipids/fly, respectively), and was not affected by sex (Tukey HSD comparisons, Figure 1C). Flies acclimated at 30°C accumulated higher lipid levels. This effect was significant for females who nearly doubled their amount of lipid reserves compared to 20 and 25°C – acclimated counterparts (462.98 ± 60.58 μg lipids/fly, Tukey HSD comparisons *p* ≤ 0.0006, Figure 1C). A similar increase in lipid content was detected for males (277.84 ± 44.30 μg lipids/fly). However, this increase was significantly lower compared to females, and different only compared to 25°C-acclimated counterparts (Tukey HSD comparisons *p* ≥ 0.037, Figure 1C).

The effect of acclimation temperature on protein reserves was also significant [*F*(2, 73) = 20.62, *p* < 0.001] and similar in males and females [acclimation temperature × sex: *F*(2, 73) = 0.50, *p* = 0.608]. Regardless of acclimation temperature, females contained significantly more protein than males [sex: *F*(1, 73) = 84.99, *p* < 0.001, Tukey HSD comparisons, Figure 1D]. Flies acclimated at 30 and 25°C contained similar protein levels, and for females, these were significantly elevated compared to the protein content of 20°C-acclimated counterparts (390.08 ± 13.17, 380.99 ± 11.34, and 310.08 ± 11.99 μg proteins/fly, respectively, Figure 1D). Male protein content significantly differed between 30 and 20°C-acclimated counterparts (295.62 ± 10.09 and 233.29 ± 7.59 μg proteins/fly, respectively), and remained intermediate at 25°C (280.05 ± 9.22 μg proteins/fly, Tukey HSD comparisons, Figure 1D).

### Acclimation Effect on Survival at Low and High Relative Humidity

#### Flies Assayed Following a Short (5–10 days) Acclimation Period

**Figure 2:**
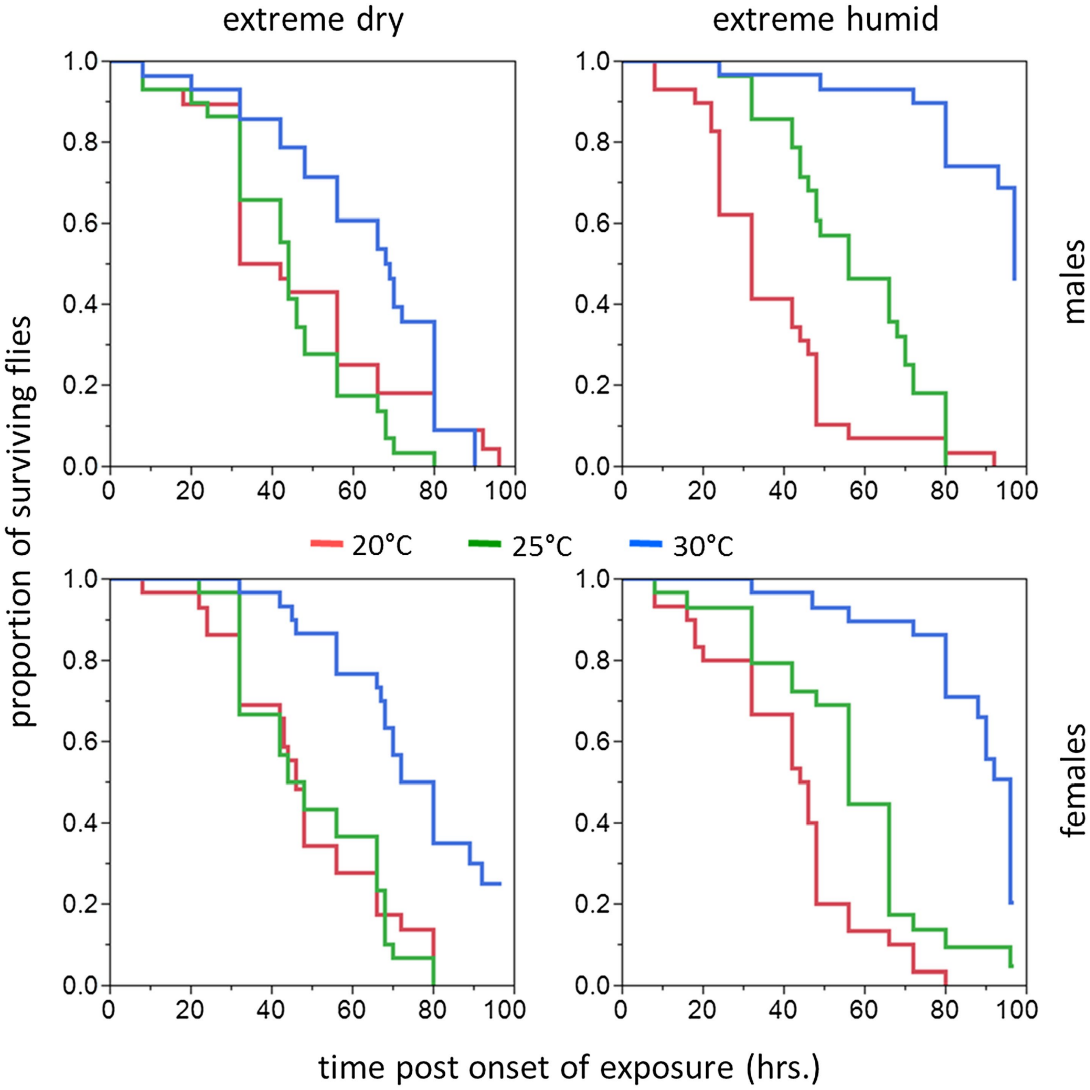
Cumulative survival plots of *B. zonata* adults exposed to extreme dry (5% RH) or extreme humid (95% RH) environments (**Left** and **Right Panel**, respectively) for 5–10 days (short acclimation period). Males (upper panels) and females (lower panels) were assayed following acclimation at 20, 25, and 30°C constant temperature regimes (*n* = 28–30 flies in each group).

**Figure 3:**
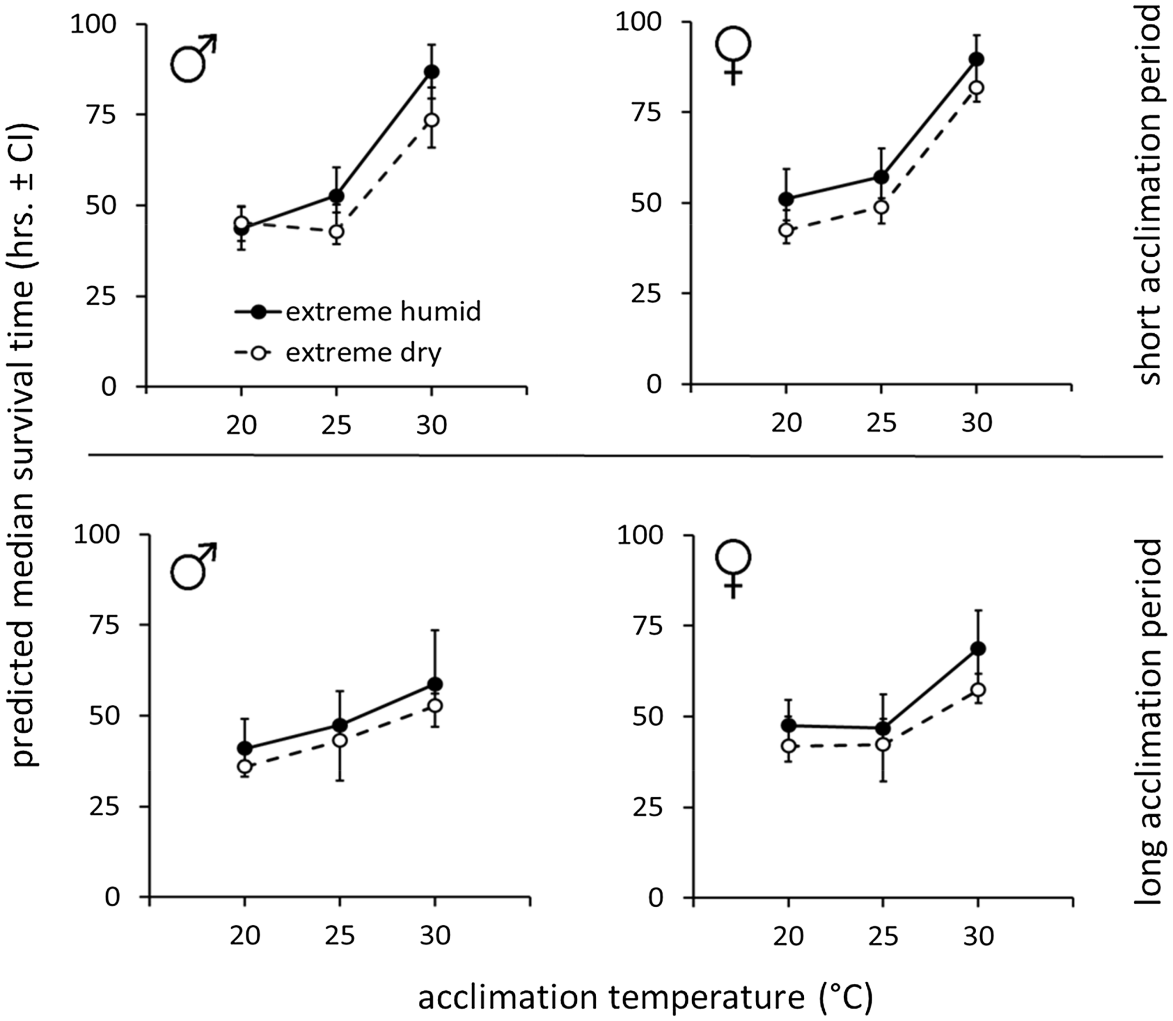
Weight-corrected median lifespan predictions (hours, ±95% CI) of *B. zonata* males and females maintained under extreme dry (5% RH) or extreme humid (95% RH) environments following acclimation at 20, 25, and 30°C for a short (5–10 days) or long (11–20 days) acclimation period (*n* = 18–30 flies in each group, total *n* = 646 flies).

Survival time of males and females acclimated at 20, 25, and 30°C significantly depended on acclimation temperature [Cox proportional hazards regression analysis: χ^2^(2) = 42.71, *p* < 0.001] and RH regimes [χ^2^(1) = 14.77, *p* < 0.001; Table 1; Figures 2, 3]. Nevertheless, sex was not a significant factor in our analysis [χ^2^(1) = 0.11, *p* = 0.738]. No significant interactions were detected between acclimation temperature, RH regime, and sex, indicating that males and females responded similarly to acclimation and RH treatments. Additionally, body weight was significantly associated with survival [χ^2^(1) = 50.68, *p* < 0.001], and the two measures were positively correlated for males and females (see Supplementary Figure S1 for pooled data excluding censored flies). No significant interactions between body weight and sex, acclimation temperature, or RH were detected, indicating that increased fresh weight similarly contributed to the life expectancy of males and females in all treatment groups.

**Table 1:** Summary of the final Cox proportional hazards regression model describing the effects of acclimation temperature, body weight, relative humidity (RH) regime, and sex on survival of adult *Bactrocera zonata* exposed to extreme dry (5% RH) or extreme humid (95% RH) environments. Survival was assayed following acclimation at 20, 25, and 30°C constant temperature regimes for a short (5–10 days) or long (11–20 days) acclimation period.

Effect	*X*^2^(df)	*p*
Short acclimation period		
Acclimation temperature	42.70 (2)	<0.001^*^
Body weight	50.68 (1)	<0.001^*^
RH treatment	14.76 (1)	<0.001^*^
Sex	0.11 (1)	0.738
Long acclimation period		
Acclimation temperature	15.76 (2)	<0.001^*^
Body weight	11.94 (1)	0.001^*^
RH treatment	11.84 (1)	0.001^*^
Sex	0.04 (1)	0.827

Effects denoted with (*) are statistically significant.

Accordingly, weight-corrected predictions of survival time indicated that males and females gained enhanced endurance and survived longer, when acclimated at 30°C, compared to counterparts maintained at 20 or 25°C (Figure 3, see Table 2 for means ± SE values). Temperature similarly affected survival at the two RH treatments. Nevertheless, flies essayed under 5% RH survived for shorter periods compared to counterparts assayed under 95% RH regardless of acclimation temperature (Figure 3; Table 2).

**Table 2:** Predicted median survival times of males and females exposed to extreme dry (5% RH) or extreme humid (95% RH) environments following acclimation at 20, 25, and 30°C constant temperature regimes for a short (5–10 days) or long (11–20 days) acclimation period. Values represent estimated marginal means (± CI) adjusted for the mean weight of the flies and are depicted in Figure 3.

Acclimation temp. (c)		Survival at 5% RH			Survival at 95% RH		
		Median time (h)	Lower 95% CI	Upper 95% CI	Median time (h)	Lower 95% CI	Upper 95% CI
Short acclimation period							
20	Females	42.48	38.76	48.03	51.13	45.10	59.40
25		48.97	44.30	55.62	57.24	51.24	64.93
30		81.93	77.87	90.38	89.62	83.41	96.24
20	Males	45.21	40.07	49.81	43.66	37.86	49.52
25		42.79	39.24	50.28	52.71	48.11	60.46
30		73.57	65.89	82.54	86.97	79.52	94.37
Long acclimation period							
20	Females	41.83	37.59	49.97	47.53	42.67	54.67
25		42.30	32.00	49.35	46.79	42.68	56.00
30		57.38	53.76	67.44	68.83	61.72	79.32
20	Males	35.97	33.10	41.07	40.95	37.50	49.15
25		43.22	32.00	48.39	47.42	43.74	56.84
30		52.79	46.97	56.00	58.75	54.07	73.59

Hazard ratios calculated by the model corroborated these effects and indicated that the risk of death for flies acclimated at 30°C (pooled males and females at both RH regimes) significantly decreased by 63.1 and 65.8% compared to 20°C [*HR* (95% CI) = 0.342 (0.242, 0.485), *p* < 0.001] or 25°C – acclimated counterparts [*HR* (95% CI) = 0.369 (0.265, 0.512), *p* < 0.001]. Acclimation at 25°C did not reduce the risk of death [0.927 (0.708, 1.215), *p* = 0.584] compared to 20°C. Additionally, the risk of death for flies assayed at 95% RH (pooled males and females from all acclimation temperatures) was significantly reduced by 37.4% compared to counterparts maintained at 5% RH [*HR* (95% CI) = 0.626 (0.492, 0.795), *p* < 0.001].

#### Flies Assayed Following a Long (11–20 Days) Acclimation Period

**Figure 4:**
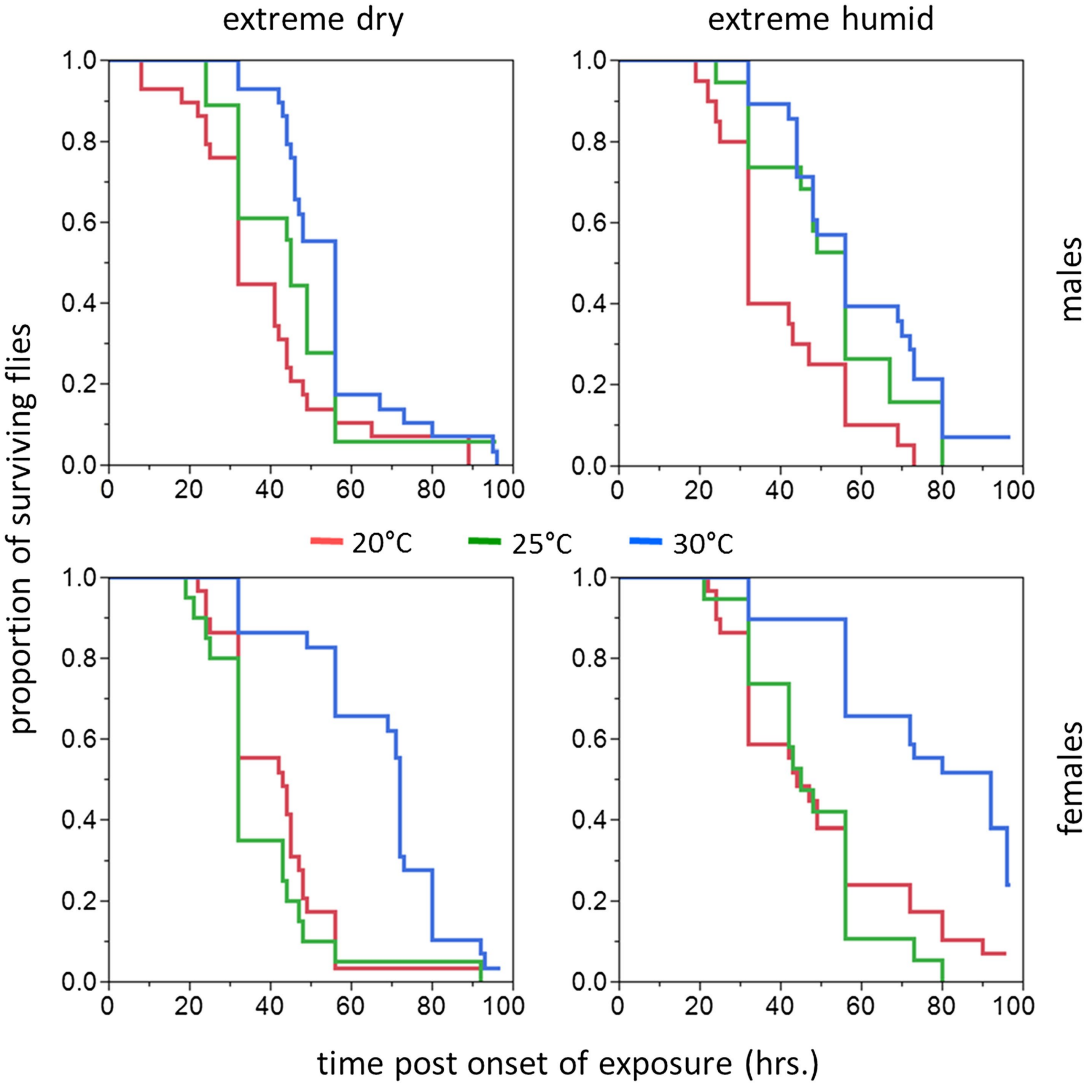
Cumulative survival plots of *B. zonata* adults exposed to extreme dry (5% RH) or extreme humid (95% RH) environments (**Left** and **Right Panel**, respectively) for 11–20 days (long acclimation period). Males (upper panels) and females (lower panels) were assayed following acclimation at 20, 25, and 30°C constant temperature regimes (*n* = 18–29 flies in each group).

Similarly to counterparts acclimated for a short period, the life expectancy of 11–20 day acclimated males and females was significantly affected by acclimation temperature [χ^2^(2) = 15.76, *p* < 0.001] and RH regime [χ^2^(1) = 11.84, *p* = 0.001]. Here also, survival times were not affected by sex [χ^2^(1) = 0.04, *p* = 0.827, Table 1; Figures 3, 4]. Body weight significantly contributed to life expectancy [χ^2^(1) = 11.94, *p* = 0.001] and was positively correlated with male and female survival (see Supplementary Figure S1 for pooled data excluding censored flies). Additionally, no significant two or three way interactions were detected indicating that male and female survival was similarly affected by weight, temperature, and RH regime. Accordingly, weight-corrected lifespan predictions of males and females were generally elevated when flies were acclimated at 30°C, compared to counterparts maintained at 20 and 25°C (Figures 3, 4; Table 2). However, differences appeared to be smaller when compared to counterparts acclimated for a short period of 5–10 days. Additionally, survival at low RH tended to be lower for both males and females at all acclimation temperatures (Figure 3; Table 2).

Hazard ratios assigned significant effects to acclimation temperature and RH regime. The risk of death was 45.5–42.4% lower for flies acclimated at 30°C (pooled males and females at both RH regiems) compared to counterparts maintained at 20°C [*HR* (95% CI) = 0.544 (0.392, 0.754), *p* < 0.001], or 25°C [*HR* (95% CI) = 0.576 (0.417, 0.796), *p* < 0.001]. The risk of death for 20 and 25°C-acclimated flies was similar [*HR* (95% CI) = 0.943 (0.693, 1.283), *p* = 0.713]. Additionally, the risk of death for flies challenged with 95% RH (pooled sexes at all acclimation temperatures) was 32.8% lower compared to flies challenged with 5% RH [*HR*(95% CI) = 0.658 (0.518, 0.835), *p* < 0.001].

Overall, our results point that regardless of acclimation period, flies gain weight according to the temperature at which they are maintained. Similarly, the buildup of lipid and protein reserves is affected by temperature, and acclimation at 30°C resulted in significantly elevated values of all of these measures. After correcting for the positive effect of weight on survival, overall lifetime predictions of males and females were found to be prolonged following acclimation at 30°C. This effect was consistent regardless of acclimation period or the ambient humidity at which the flies were maintained. Finally, we found that survival time was reduced in the low humidity environment.

## Discussion

### Effect of Acclimation Temperature

Acclimation at constant temperature regimes had a profound effect on the survival of *B. zonata* in our experiments (Table 1; Figure 3). For both males and females, preconditioning at 30°C resulted in higher survival rates at both low and high humidity regimes compared to counterparts acclimated at 20 and 25°C. During these bioassays, flies were subjected to contrasting RH regimes while simultaneously deprived of food, thus experiencing stress from desiccation and starvation alike (a widely used setting for testing desiccation resistance in *Drosophila*, Gibbs et al., 1997). Thus, the exact mechanisms triggered by temperature and which contributed to survival in our experiments are difficult to pinpoint. Nevertheless, the difference in mortality rates in the two RH regimes suggests that under high RH, where intrinsic water is retained more readily, flies eventually died of starvation. Conversely, in the desiccating environment, acute water loss may have preceded or complemented the depletion of nutritional reserves and eventually resulted in higher mortality rates.

The contribution of acclimation at 30°C to survival at both RH regimes suggests that a similar mechanism acted in both cases. Our results point that weight, associated with acclimation temperature may be involved. Flies maintained for 5–10 days at 30°C were 33 and 19.7% heavier compared to flies acclimated at 20 and 25°C (respectively). Similarly, flies maintained at 30°C for 11–20 days were 25.7 and 12.7% heavier compared to 20 and 25°C – acclimated counterparts. This gain in weight was positively and significantly correlated with the survival of the flies (Table 1; Supplementary Figure S1). Accordingly, we suggest that the elevated survival rates of 30°C-acclimated flies resulted, at least partly, from temperature-mediated gain in weight. Although not quantified in our study, foraging activity seemed to be modulated by temperature, and was encouraged in flies maintained at 30°C (D. Nestel, personal observations). Thus, increased feeding activity and possibly accelerated metabolism probably accounted for the recorded weight gain and contributed to survival following acclimation at 30°C.

Similar effects of acclimation temperature on weight gain were reported for other insects as well (Weldon et al., 2013; Fischer and Kirste, 2018), and weight was previously associated with increased tolerance to desiccation in some tephritids (Weldon et al., 2013, 2016). However, in other cases, the correlation between body weight and survival under different temperature and RH regimes was uncertain (e.g., *C. capitata*, Weldon et al., 2018). Our analysis suggests that weight gain affected survival only indirectly, possibly through temperature-dependent accumulation of nutritional reserves. We base this conclusion on the following observations: first, the analysis we employed estimated weight-adjusted survival times to be higher in flies acclimated at 30°C (Figure 3), suggesting that temperature, in addition to weight-gain contributed to increased survival. Secondly, survival was not affected by sex although females were generally heavier than males. Finally, our analysis reveals similar survival patterns in flies acclimated for 5–10 days and 11–20 days, regardless of increased weight of the latter (Figures 1A,B). This suggests that body weight may be an indirect predictor of survival and highlight the possibility that accumulation of nutritional reserves mediated by temperature were responsible for prolonging survival in our study.

In this regard, lipids or other nutritional reserves (e.g., glycogen) may have been involved in mediating survival under stress. Lipids and glycogen are used as an energy source during starvation and their catabolism yields substantial amounts of water as a metabolic byproduct, thus contributing to desiccation resistance as well (Gibbs et al., 1997; Arrese and Soulages, 2010). The association between lipid utilization and enhanced survival during starvation and desiccation resistance assays applies for fruit flies as well (e.g., Tejeda et al., 2014; Weldon et al., 2016). According to our study, males and females acclimated at 30°C accumulated significantly higher lipid levels then counterparts maintained at 20 and 25°C (Figure 1C). Their resistance to starvation and desiccation was thus probably better supported by these resources. Nevertheless, females contained significantly more lipids than males, which may be related to the accumulation of lipoprotein during egg maturation (e.g., Haq et al., 2010; Yadav and Singh, 2018). Higher lipid loads in females could have been expected to increase their survival compared to males. However, this situation was not observed. Possibly, some of these reserves remain inaccessible once eggs are produced, excluding the possibility of egg-reabsorption and use of lipids for maintenance (Aluja et al., 2011). Thus, although, we do not measure lipid allocation for reproduction and maintenance, it is possible that the load of lipid reserves aimed at maintenance are similar in both sexes, resulting in similar survival patterns of males and females. These possibilities require further studies.

Similarly to lipids, protein content was also associated with acclimation temperature, but in this case, flies maintained at 25 and 30°C accumulated similar levels of protein (Figure 1D). These results, together with other studies suggest that protein reserves contribute negligibly to survival under starvation and desiccation stress (see Weldon et al., 2016, 2019). Taking it altogether, we assume that accumulation of nutritional reserves (possibly lipids) resulting from acclimation at higher temperatures, promoted the tolerance of *B. zonata* to starvation and desiccation in our study.

### Effect of RH

Survival was significantly reduced under the low RH regime, indicating that desiccation was a significant stress factor for the flies in our experiments. Additionally, acclimation at 30°C prolonged survival under low RH conditions.

Increased resistance to desiccation can potentially be achieved through efficient water management. In this context, three mechanisms have been suggested: increased water storage (e.g., by increased body size or lipid catabolism), ability to reduce water loss (e.g., by respiratory adaptations or excretion of cuticular lipids), and increased tolerance to water loss through activation of stress-induced osmoregulatory mechanisms (reviewed by Weldon et al., 2019). One or more of these mechanisms may have acted either alone or in conjunction to support the survival of *B. zonata* under low RH in our experiments. However, if acclimation temperature had affected any of these mechanisms, we would expect a significant interaction between acclimation temperature and RH treatment (which was not detected). Accordingly, as it stands we cannot provide evidence as to the effect acclimation temperature through such mechanisms in our experiment. Nevertheless, the fact that acclimation at 30°C similarly affected survival in the two RH regimes suggest that it acted mainly through increasing resistance to starvation.

### Effect of Sex

Contrary to acclimation temperature and humidity, sex was not a significant predictor of survival in our dataset (Table 1; Figure 3). This finding contrasts with studies on other tephritids, such as *Bactrocera. tryoni* (Froggatt; Weldon and Taylor, 2010; Weldon et al., 2013), *Anastrepha ludens* (Loew; Tejeda et al., 2014), and *Ceratitis capitata* (Wiedmann; Weldon et al., 2016) highlighting males as more tolerant to desiccation compared to females. Nevertheless, these sex-related differences in desiccation resistance seem to depend on temperature, age, and origin of the flies, and may be reversed in other species (see Matzkin et al., 2009; Weldon and Taylor, 2010; Weldon et al., 2013, 2016). For example, female tolerance to desiccation may be related to partitioning of water resources between egg production and soma maintenance, i.e., that water contained in eggs remain inaccessible to gravid females, thus, reducing their tolerance to low humidity (Weldon et al., 2013, 2016). If females in our experiments were not yet producing eggs (sexual maturity in our *B. zonata* colony takes more than 15 days), they were probably able to sequester available water and survive similarly to males. Alternatively, if eggs were being produced the partitioning of lipids between maintenance and reproduction may have played part in determining survival (as discussed above). In any case, differences in male and female survival seem to be conditional on the context of the experimental setting and involved species, and need to be interpreted accordingly.

### Effect of Acclimation Period

Although we do not compare flies of different acclimation periods, our results point that survival was similarly affected by temperature and humidity regardless of the flies preconditioning duration. Thus, a short acclimation period of 5–10 days at 30°C had a similar effect as a long acclimation, and was sufficient to prolong survival in our experiments. Nevertheless, flies acclimated for a long period were generally less tolerant and survived for shorter periods compared with younger counterparts. This seems to apply particularly for males (Figure 3), and may be related to the older age of the flies. Indeed, age was found to significantly affect survival under starvation and desiccation stress in other tephritids (Weldon and Taylor, 2010), as well as in Drosophilla (Gibbs and Markow, 2001). In the latter case, age was associated with greater rates of water loss leading to mortality under desiccating conditions. A decline in desiccation resistance in older flies was previously suggested to associate with allocation of resources to support reproductive organs or sexual activity (see Weldon and Taylor, 2010). Our experiments do not allow for isolating specific factors contributing to possible age-associated differences in survival.

### Thermal Acclimation and Tolerance to Humidity Stress

Regardless of the strong climatic association between temperature and humidity, the effect of thermal acclimation on tolerance to humidity stress has been poorly studied in insects (Terblanche and Hoffmann, 2020). The few studies conducted up to date have shown, even within the same species, an inconsistent and varied desiccation resistance response resulting from adaptive phenotypic plasticity to thermal acclimation (Fischer and Kirste, 2018). For example, while thermal treatment during exposure to hydric stress of a laboratory strain of *C. capitata* affected tolerance to humidity stress (Weldon et al., 2016), field collected populations from different mesic and xeric environments with diverse thermal regimes, showed inconsistent tolerance to hydric stress (Weldon et al., 2018). Similarly, different species of tsetse flies derived from laboratory colonies showed diverse and complex water balance responses to humidity stress when exposed to various combined temperature and humidity conditions (Kleynhans and Terblanche, 2011). In some cases, the resulting responses, both in *C. capitata* and tsetse flies, contrasted with the response predicted from the geographic occupancy of the species (i.e., species derived from xeric or mesic environments; Kleynhans and Terblanche, 2011; Weldon et al., 2018). The observed variability may point to different laboratory methodologies followed to explore acclimation effects on the tolerance to extreme climatic conditions, or the effect of genotypic variability within and between populations (Terblanche and Hoffmann, 2020). In case of the present study, we used a laboratory colony, which probably have a reduced genetic variability and thus express a relatively uniform physiological response to temperature acclimation and RH. We also applied a uniform experimental protocol to all the assayed individuals, minimizing random effects resulting from genetic and methodological factors. Our results clearly show that acclimation at 30°C positively affect the survival of *B. zonata* in extreme high and low humidity conditions. We assume that temperature acted by modulation of feeding and metabolism, resulting in accumulation of weight and nutritional reserves. Consequently, survival was affected mainly through the increase of the flies resistance to starvation.

### Summary

Native to the humid tropics of South and South-East Asia (EPPO Bulletin, 2010), *B. zonata* has dispersed to North Africa and east Mediterranean (EPPO Bulletin, 2010) regions characterized by dry climatic conditions. Given its apparent adaptability to humidity stress in its habitat, the species has a large potential to expand its range further into Mediterranean Europe, where it is considered a type A1 pest by the European and Mediterranean Plant Protection Organization (EPPO Bulletin, 2010), and to other tropical and subtropical areas of the World (Ni et al., 2012). Herein, we demonstrate that thermal acclimation contribute to desiccation resistance of this fly. Our results suggest that acclimation temperature indirectly affect survival through modulation of feeding and accumulation of nutritional reserves. Accordingly, we highlight the conditioning of metabolism as a major link between thermal acclimation and desiccation resistance. This study, thus, set the basis to understand the current geographic range of the species from the hydric perspective. The thermal limits of its expansion still need to be clarified and studied in order to produce more accurate risk maps and prepare preventive actions to reduce risk of introduction. This evaluation is currently underway in our laboratory.

## Data Availability Statement

The raw data supporting the conclusions of this article will be made available by the authors, without undue reservation.

## Author Contributions

DN conceived and planned the study. YA and EN-L executed the experiments. MB-Y and EV analyzed the data. DN, MB-Y, NP, and EV contributed to writing and approval of the manuscript. All authors contributed to the article and approved the submitted version.

## Conflict of Interest

The authors declare that the research was conducted in the absence of any commercial or financial relationships that could be construed as a potential conflict of interest.

## Publisher’s Note

All claims expressed in this article are solely those of the authors and do not necessarily represent those of their affiliated organizations, or those of the publisher, the editors and the reviewers. Any product that may be evaluated in this article, or claim that may be made by its manufacturer, is not guaranteed or endorsed by the publisher.
