# Supplementary material for: Effects of Thermal Acclimation on the Tolerance of Bactrocera zonata (Diptera: Tephritidae) to Hydric Stress
Source: Front Physiol. 2021 Sep 3;12:686424. doi: 10.3389/fphys.2021.686424 (PMC8446596; doi:10.3389/fphys.2021.686424)

Effects of thermal acclimation on the tolerance of Bactrocera zonata (Diptera: Tephritidae) to hydric stress

Supplementary figure S1. Association (Pearson correlation) between survival time and fresh weight of *Bactrocera zonata* female (red circles) and male (blue circles) acclimated for 5-10 days or 11-20 days (short and long acclimation periods, respectively) at three different acclimation temperatures. Censored observations corresponding with flies who remained alive at the end of the assays and included in our survival models (Fig. 4), were omitted from this analysis.

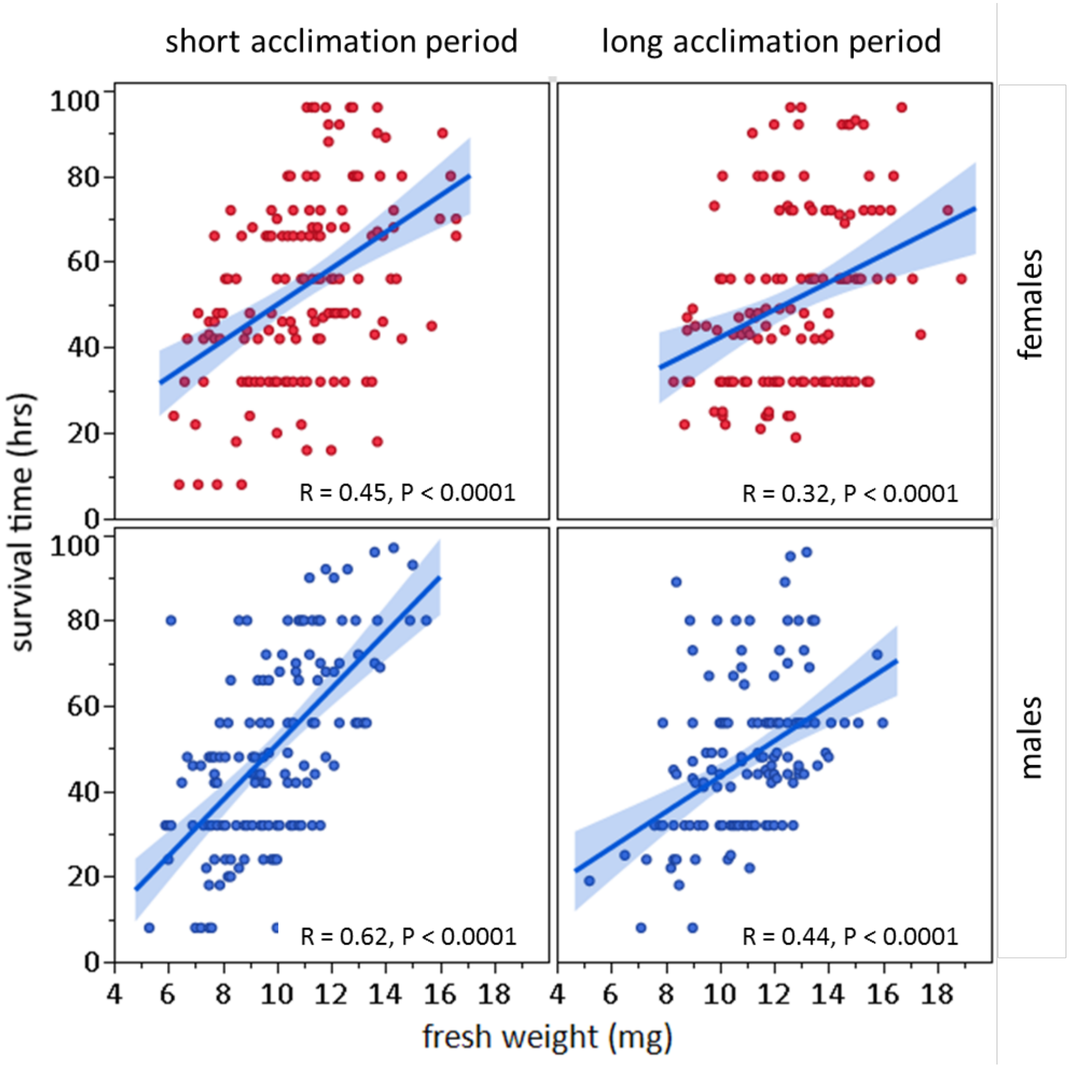

Supplement: Supplementary file 1 [file Image_1.pdf]
